# CD8^+^ T-cell recognition of a synthetic epitope formed by *t*-butyl modification

**DOI:** 10.1111/imm.12398

**Published:** 2015-03

**Authors:** Reiss A Reid, James E Redman, Pierre Rizkallah, Chris Fegan, Chris Pepper, Stephen Man

**Affiliations:** 1 School of Medicine, Institute of Cancer and Genetics, Cardiff UniversityCardiff, UK; 2 School of Chemistry, Cardiff UniversityCardiff, UK; 3 School of Medicine, Institute of Infection & Immunity, Wales Heart Research Institute, Cardiff UniversityCardiff, UK

**Keywords:** CD8^+^ T cells, mass spectrometry, peptides

## Abstract

We set out to clone Bax-specific CD8^+^ T cells from peripheral blood samples of patients with primary chronic lymphocytic leukaemia. A number of clones were generated using a Bax peptide pool and their T-cell epitope was mapped to two peptides sharing a common 9-amino-acid sequence (LLSYFGTPT), restricted by HLA-A*0201. However, when these T-cell clones were tested against highly purified syntheses (> 95%) of the same peptide sequence, there was no functional response. Subsequent mass spectrometric analysis and HPLC fractionation suggested that the active component in the original crude peptide preparations (77% pure) was a peptide with a *tert*-butyl (*t*Bu) modification of the tyrosine residue. This was confirmed by modification of the inactive wild-type sequence to generate functionally active peptides. Computer modelling of peptide:HLA-A*0201 structures predicted that the *t*Bu modification would not affect interactions between peptide residues and the HLA binding site. However, these models did predict that the *t*Bu modification of tyrosine would result in an extension of the side chain out of the peptide-binding groove up towards the T-cell receptor. This modified product formed < 1% of the original P603 crude peptide preparation and < 0·05% of the original 23-peptide mixture used for T-cell stimulation. The data presented here, illustrate the potential for chemical modifications to change the immunogenicity of synthetic peptides, and highlight the exquisite capacity of T-cell receptors to discriminate between structurally similar peptide sequences. Furthermore, this study highlights potential pitfalls associated with the use of synthetic peptides for the monitoring and modulating of human immune responses.

## Introduction

Synthetic peptides containing immunogenic T-cell epitopes have been widely used in various aspects of immunotherapy from adoptive cell transfer^1^ to peptide vaccination.^2^ This approach provides a cost effective and reliable source of antigens that can be synthesized to suitable quantities and purity grades. This process eliminates the introduction of potential virulence factors, which may occur with the use of recombinant viral vaccines. Furthermore, the nature of the chemical synthesis process allows for peptides to be modified to exacting specifications that can enhance their MHC binding capacity, their *in vivo* stability^3^ and ultimately their immunogenicity.^2^,^4^

Historically mixtures of synthetic peptides containing multiple epitopes originating from specific proteins have been used to measure human memory T-cell responses against viruses such as human cytomegalovirus,^5^ Epstein–Barr virus^6^ and human papillomavirus.^7^ Through the use of smaller and more refined peptide mixtures it is possible to map the precise epitope specificity of individual T-cell clones.^8^ Such epitopes can then be incorporated into tetramer reagents to allow direct *ex vivo* measurement of memory T cells in response to natural infection or vaccination.^9^

In a previous study, we used pooled synthetic peptide mixtures as immunogens to generate human T cells (from healthy donors) against candidate tumour antigens *in vitro*. This approach allowed us to test the concept that Bax, a pro-apoptotic protein that is abnormally degraded in human cancers, can generate T cells with activity against primary human cancer cells.^8^

In the current study, we used the same approach, but using blood from patients with chronic lymphocytic leukaemia (CLL) in an attempt to generate Bax-specific T-cell clones. We used a combined immunological (for the assessment of T-cell function and response) and physicochemical (for the identification and characterization of peptides) approach to map a human CD8^+^ T-cell clone specificity to a novel synthetic epitope. This finding highlights the immunogenicity of chemically modified peptides and has implications for the use of synthetic peptides to generate tumour-specific T cells.

## Materials and methods

### Blood samples

Healthy volunteer blood samples were collected locally and CLL samples were derived from clinics at the University Hospital of Wales and Llandough Hospital. All samples were collected with informed consent with ethical approval [South East Wales Research Ethics Committee (02/4806)]. Peripheral blood mononuclear cells were isolated by Histopaque-1077 (Sigma-Aldrich, Poole, UK) centrifugation, as previously described.^10^

### Induction of Bax-specific T cells

CD8^+^ T cells were immunomagnetically enriched from peripheral blood mononuclear cells using anti-human CD8 microbeads according to the manufacturer’s instructions (Miltenyi Biotec, Woking, UK). After enrichment, the T cells were cultured at 37°/5% CO_2_ for 48 hr in AB-RPMI medium supplemented with interleukin-7 (IL-7; 10 ng/ml; Peprotech, London, UK) to allow for the activation of autologous CLL cells to be used as a source of antigen-presenting cells. AB-RPMI consisted of RPMI-1640 (Sigma-Aldrich) supplemented with 5% human AB serum, glutamine (2 mmol/l), streptomycin (100 μg/ml), penicillin (100 U/ml), HEPES (25 mmol/l) (all sourced from Life Technologies, Paisley, UK). After 48 hr the T cells were harvested, washed and cultured in the presence of irradiated activated autologous CLL cells at a 4 : 1 ratio in AB-RPMI supplemented with Bax peptide pool (10 μg/ml; Mimotopes, Clayton, Victoria, Australia)^8^ and IL-2 (40 U/ml; Proleukin, Novartis, Frimley, UK). After 3 days, 500 μl of AB-RPMI supplemented with IL-2 (120 U/ml) and IL-7 (30 ng/ml) was added. The cultures were re-stimulated weekly with the peptide pool and autologous activated CLL cells. On day 35 the T cells were harvested and Bax peptide immunogenicity was tested by ELISpot assay.

### Isolation and cloning of peptide-specific T cells

Peptide specific interferon-*γ* (IFN-*γ*) production was induced after culturing the polyclonal T cells with the Bax peptide pool (10 μg/ml) presented on autologous activated CLL cells for 5 hr at 37°/5% CO_2_. Interferon-*γ* secreting T cells were immunomagnetically enriched using anti-human IFN-*γ* beads according to the manufacturer’s protocol (Miltenyi Biotec). The isolated T cells were ‘rested’ overnight in AB-RPMI supplemented in IL-2 (40 U/ml) and IL-7 (10 ng/ml) before cloning by limiting dilution as previously described.^8^

### Measurement of IFN-*γ* release

For IFN-*γ* ELISA, T cells (1 × 10^5^) were cultured in 200 µl of AB-RPMI at a 1 : 1 ratio with peptide-pulsed T2 cells for 18 hr in U-bottomed tissue culture plates. T cells were also cultured with unpulsed T2 cells (negative control) or mitogen (positive control – phytohaemagglutinin, 10 µg/ml, P1585 – Sigma Aldrich). Cell-free supernatants were harvested and analysed by ELISA for human IFN-*γ* (Human IFN-*γ* ELISA^PRO^ kit; Mabtech, Nacka Strand, Sweden).

Interferon-*γ* ELISpots were performed as previously described.^7^ Briefly, T cells were plated in triplicate at 1 × 10^5^ (initial screen) or 1 × 10^4^ to 3 × 10^4^ cells (clones/lines) per well in MultiScreen HTS IP Filter Plates (Millipore, Watford, UK). T cells were cultured at 1 : 1 ratio with T2 cells ± Bax peptides (10 μg/ml). T cells were also incubated in the absence of T2 cells (negative control) or with mitogen (positive control). The plates were developed using the AP Conjugate substrate kit (BioRad, Hemel Hempstead, UK). The numbers of spots/well were counted with an ELISpot reader (AID, Oxford Biosystems Cadama, Wheatley, Oxfordshire, UK). Specific peptide responses were calculated by subtracting the background response (T cells + T2) from the T cells + T2 + peptide wells.

For IFN-*γ* ELISA intracellular cytokine staining, T cells (1 × 10^5^) were cultured in AB-RPMI at 1 : 1 ratio with T2 cells ± peptide in the presence of GolgiStop™ and GolgiPlug™ (BD, Oxford, UK). T cells were also cultured in the presence of mitogen (positive control). After 5 hr the cells were washed and co-stained with anti-human CD3*-*FITC (Biolegend, London, UK – clone UCHT1) and CD8-peridinin chlorophyll protein (Biolegend – clone HIT8a). The cells were then fixed and permeabilized with Leucoperm™ (AbD Serotec, Kidlington, UK) and intracellular IFN-*γ* was identified using anti-human IFN-*γ*-phycoerythrin (eBioscience, Hatfield, UK – clone 4S.B3). The cells were analysed using an Accuri C6 (BD) and data analysis was performed using cflow software (BD).

### CD107*α* surface staining

T cells (1 × 10^5^) were cultured in AB-RPMI at 1 : 1 ratio with T2 cells ± peptide in the presence of GolgiStop™ and GolgiPlug™ (BD). T cells were also cultured in the presence of mitogen (positive control). Changes in the surface expression of CD107*α* were determined through the addition of anti-human CD107*α*-phycoerthrin (Biolegend – clone H4A3) to each culture. After 5 hr the cells were washed and co-stained with anti-human CD3-FITC (Biolegend – clone UCHT1) and CD8-peridinin chlorophyll protein (Biolegend – clone HIT8a). The cells were analysed using an Accuri C6 (BD) and data analysis was performed using cflow software (BD).

### Peptides

Twenty-three candidate peptides were identified from the amino acid sequence of Bax using predictive computer algorithms for HLA binding, as previously described.^8^ Aliquots from these stock peptides were pooled (Bax pool 601–23) and stored at −80°. Smaller pools of five or six peptides were made for epitope mapping. Highly purified (> 95%) Bax P603 peptides were synthesized (ProImmune, Oxford, UK and Peptide Synthetics, Bishops Waltham, UK). Highly purified (> 98%) Bax P603 *t*Bu peptide was synthesized using *t*-butyl-modified tyrosine (PolyPeptide Group, Strasbourg, France).

### Peptide analysis

Liquid chromatography mass spectrometry (LCMS) was performed on an Ultimate 3000 (Dionex, Sunnyvale, CA) HPLC system interfaced to an amaZon SL ion trap spectrometer (Bruker, Billerica, MA). Chromatographic separation was performed with a 100 × 2·1 mm Ace Excel 2 column at a flow rate of 0·5 ml/min, eluting with a binary gradient of acetonitrile/water (0·1% formic acid). The column was equilibrated for 5 min with 5% acetonitrile, followed by a linear gradient to 95% acetonitrile over 20 min. MS^2^ spectra were collected using collision-induced dissociation with automatic or manual precursor selection. Specific single amino acid deletion peptides were identified using multiple reaction monitoring of each of the corresponding [M + H]^+^ precursor ions.

### Peptide library synthesis

Peptides were synthesized manually on Rink Amide methylbenzhydryl amine resin (Novabiochem, Watford, UK) using standard fluorenylmethyloxycarbonyl (Fmoc) protocols, as previously described.^11^ To prepare the libraries, the resin was split into three 50 mg portions (1–3) and single amino acid deletions were introduced into the C-terminal residues (GTPT, portion 2) and the N-terminal residues (LSYF, portion 3). After coupling of the sequence LLSYFGTPT (portion 1), a 20 mg peptide-resin sample was withdrawn and subjected to a further deprotection-coupling cycle with FmocLeu-OH to obtain LLLSYFGTPT (portion 4).

### Fractionation of peptide

Fractionation of LLSYFGTPT-NH_2_ (portion 1) was performed by HPLC (Waters 2525 pump and 2996 detector; Waters Corporation, Milford, MA) with a Vydac 218TP 250 × 22 mm column. Peptides were eluted with a binary gradient of 0 min 95% A/5% B, 1 min 95% A/5% B, 16 min 50% A/50% B where solvent A = H_2_O, 0·05% trifluoroacetic acid (TFA) and solvent B = acetonitrile, 0·05% TFA at a flow rate of 22·9 ml/min. The principal peak eluted at 11·5 min, with further peaks collected at 9·1, 9·4, 10·0, 10·4, 13·9, 14·4 and 15·3 min.

### Modification of wild-type P603 peptide

An aliquot of P603 (Peptide Synthetics, 95% Pure) in DMSO was added to diethyl ether and shaken vigorously to precipitate the peptide. Subsequently the precipitated peptide was recovered by centrifugation, washed with diethyl ether and dried *in vacuo*. The peptide was treated under different conditions to introduce *t*Bu groups into the wild-type (wt) peptide sequence.

Briefly, P603 peptide was added to a solution of di-*tert*-butyl dicarbonate (12 mg) in CHCl_3_. The CHCl_3_ was evaporated by a stream of N_2_, then TFA (1 ml), triisopropylsilane (25 µl) and water (25 µl) were added. After 1 hr, the solution was concentrated by N_2_ blowing to approximately 50% of its original volume, then pipetted into diethyl ether (14 ml). Precipitate was collected by centrifugation, washed twice with diethyl ether and allowed to dry in air.

P603 was also dissolved in TFA (1 ml) and methylpropene (Sigma-Aldrich) was bubbled through the solution for 15 min. The solution was blown with a stream of N_2_ to evaporate volatile material and extracted with water (14 ml). The aqueous extract was concentrated by rotary evaporation then lyophilized.

### MHC:peptide model preparation

The starting model was based on entry 4I4W^12^ from the Protein Data Bank (http://www.rcsb.org). COOT^13^ was used to modify the sequence of the peptide to be LLSYFGTPT. The conformation of the peptide was restricted to be as close as possible to that in 4I4W. For the purpose of the present work, this model was referred to as ‘wild type’, or wt. Y4 of the peptide was modified by adding a *t*Bu group at the meta position of the aromatic ring of the tyrosine side-chain. As this ring can rotate between two positions around the χ^2^ dihedral, two possible conformations need to be considered (Fig.6). The geometry of the Tyr(3-*t*Bu) was regularized by applying the geometry restraints generated with JLIGAND.^14^ PYMOL was used to generate the graphical representations in Fig.7.

## Results

### Detection and isolation of Bax peptide-specific T cells

**Figure 1:**
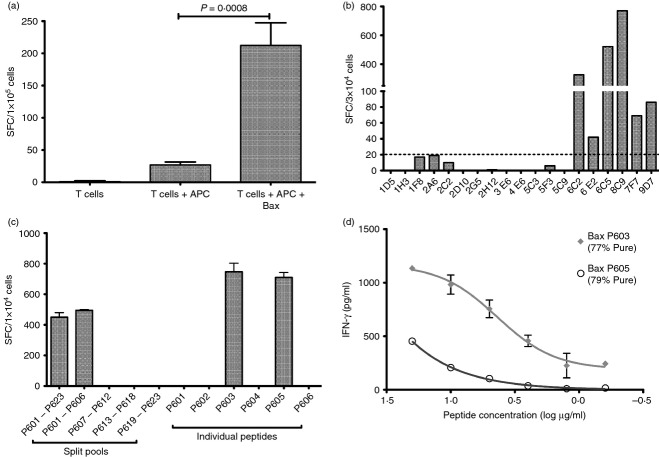
T-cell responses against Bax peptides and mapping the specificity of the CD8^+^ T-cell clone 6C5. (a) Purified CD8^+^ T cells were cultured with irradiated autologous activated B-CLL cells and Bax peptides 601–23 for 5 weeks before testing by interferon-*γ* (IFN-*γ*) ELISpot. Antigen-presenting cells (APC) were autologous activation B-CLL cells. Numbers shown are spots/10^5^ T cells (mean of triplicates ± SD, *n *= 1) Statistical analysis (unpaired two-tailed *t*-test) was carried out using GraphPad Prism (GraphPad Software, Inc., La Jolla, CA). (b) T-cell cultures generated by limited dilution were tested for the recognition of Bax peptides 601–23 by IFN-*γ* ELISpot. T cells were plated (∼2 × 10^4^ to 3 × 10^4^/well) with APC or with APC + peptides at a 1 : 1 ratio. APC were T2 cells. Background responses (T cells + T2) were subtracted from the data (*n *= 1). (c) 6C5 was tested by IFN-*γ* ELISpot against the Bax peptide pool 601–23, split pools, and individual peptides. T cells were plated (1 × 10^4^/well) in triplicate with T2 or T2 + peptides at a 1 : 1 ratio. Background response (T cells + T2) was subtracted from the data (5 SFC/10^4^ cells) (mean ± SD of triplicates, *n *= 2). (d) 6C5 was assayed against T2 cells pulsed with varying concentrations (20–6·25 μg/ml) of Bax P603 and Bax P605 at 1 : 1 ratio for 18 hr. Cell-free supernatants were harvested and tested for the presence of IFN-*γ* by ELISA. The EC_50_ value was calculated using the fitted curve, P603 – 4·92 µm and P605 > 100 µm (mean ± SD of duplicates, *n *= 3).

Our original aim was to isolate from patients with CLL CD8^+^ T-cell clones specific for peptides derived from the pro-apoptotic protein, Bax. Subsequently a Bax peptide pool (P601–623) was used to stimulate CD8^+^ T cells isolated from HLA-A*0201^+^ patients and Bax immunogenicity was assessed by IFN-*γ* ELISpot. After 5 weeks of peptide stimulation a highly significant (*P* = 0·0008) Bax-specific response was observed from patient R6A8R89 (Fig.1a).

Bax-specific CD8^+^ T cells were immunomagnetically enriched on the basis of IFN-*γ* secretion and cloned by limiting dilution. Six lines (6C2, 6E2, 6C5, 8C9, 7F7 and 9D7) exhibited positive Bax responses (> 20 spots/3 × 10^4^) and were selected for further characterization (Fig.1b). The putative T-cell clones were first tested against the full peptide pool to reaffirm Bax specificity; then against four smaller sub pools (Bax P601–606, Bax P607–612, Bax P613–618 and Bax P619–623) to narrow down the response, followed by individual peptides for epitope identification (Fig.1c). T-cell clones 6C5 and 8C9 both exhibited positive responses against the full Bax peptide pool and the sub-pool Bax P601–606. Of the peptides within the Bax P601–606 pool, only P603 and P605 induced an ELISpot response (Fig.1c). Interestingly, these two peptides shared an overlapping nine amino acid sequence: Bax P603 is a 9mer (Bax_161–169_; LLSYFGTPT) and Bax P605 is a 10mer (Bax_160–169_; GLLSYFGTPT). T-cell receptor (TCR) V*β* chain staining was performed and indicated the presence of a single V*β* chain (V*β* 13.1) in both lines, indicating clonality (data not shown). Of the two clones identified, 6C5 was selected for further characterization because of its superior growth kinetics.

Peptide dose–response experiments confirmed that 6C5 recognized both peptides (P603 and P605) but had a greater avidity for P603 as determined by comparison of the EC_50_ values, P603 = 4·92 μm and P605 > 100 μm (Fig.1d).

### 6C5 recognized crude but not highly purified P603 peptide

**Figure 2:**
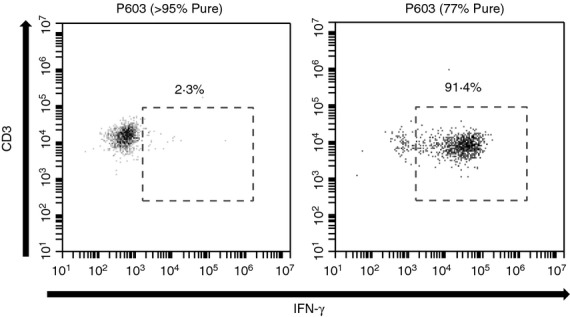
6C5 recognition of crude P603 but not highly purified P603. Representative sample of flow cytometry analysis (three independent experiments) of intracellular staining of interferon-*γ* (IFN-*γ*). T cells (1 × 10^5^/tube) were cultured in the presence of T2 or T2 + Bax peptide P603 (> 95% Pure, 10 μg/ml) or P603 (77% Pure, 10 μg/ml) at a 1 : 1 ratio for 5 hr. Lymphocytes were gated based on their forward and side scatter profile and then doublet exclusion was performed based on forward scatter height versus forward scatter width. T cells were then gated on CD3^+^ CD8^+^ cells and IFN-*γ* production was assessed through intracellular staining with anti-IFN-*γ* (mean ± SD of duplicates, *n *= 3). Statistical analysis (unpaired two-tailed *t*-test) was carried out using GraphPad Prism.

The initial results suggested that the T-cell clone 6C5 recognized the nine amino acid sequence (LLSYFGTPT) common to P603 and P605 (Fig.1c,d). As neither peptide preparation was 100% pure it was possible that the activation of 6C5 was associated with other peptide species (8mers, 10mers and modified 9mers). Therefore 6C5 was tested against highly purified P603 peptides (> 95% pure) obtained from two independent commercial sources (Proimmune and Peptide Synthetics). 6C5 failed to respond to the purified forms of P603 (95% pure), however the original P603 preparation (77% pure) induced a robust and highly significant (*P* < 0·0001) IFN-*γ* response (Fig.2). These results suggested that the immunogenicity associated with the original P603 (77% pure) was not a result of the peptide sequence (LLSYFGTPT).

### Comparison of crude and highly purified P603

Samples of crude and purified P603 peptide were analysed by ion trap LCMS (see Supporting information, Fig. S1). The samples were investigated for the presence of 8mers, 10mers and modified 9mers (Table1). Peaks corresponding to ΔF, ΔL, ΔY, ΔP, ΔT, ΔS 8mer peptides were observed in both the crude and purified samples. Fragmentation spectra of an ion of *m/z* 1111·6 and the corresponding doubly charged ion were consistent with a peptide carrying an additional N-terminal leucine residue, LLLSYFGTPT. An ion of *m/z* 1054·6 was assigned as a reaction product of the tyrosine side chain with tertiary butyl cations liberated during the final deprotection and cleavage of the peptide.

**Table 1:** Comparison of purified and crude P603 peptide samples by liquid chromatography mass spectrometry

Peptide	r.t. (min)	Transition	Crude P603	Purified P603
LLSYGTPT	5·5	851·5 → 617·3 (b_6_−H_2_O)	✓	✓
LSYFGTPT	6·3	885·4 → 651·3 (b_6_−H_2_O)	✓	✓
LLSFGTPT	6·8	835·5 → 601·3 (b_6_−H_2_O)	✓	✓
LLSYFGTT	7·0	901·5 → 764·4 (b_7_−H_2_O)	✓	✓
LLSYFGTPT	7·1	998·5 → 764·4 (b_7_−H_2_O)	✓	✓
LLSYFTPT	7·1	941·5 → 707·4 (b_6_−H_2_O)	✗	✓
LLSYFGTP	7·2	897·5 → 764·4 (b_7_−H_2_O)	✓	✗
LLYFGTPT	7·4	911·5 → 677·4 (b_6_−H_2_O)	✓	✓
LLLSYFGTPT	8·0	1111·6 → 877·5 (b_8_−H_2_O)	✓	✗
LLSY(3-*t*Bu)FGTPT	9·3	1054·6 → 820·5 (b_7_−H_2_O)	✓	✗

Chromatography was performed on an Ace Excel 2 100 × 2·1 mm column (C18), eluting with a linear gradient of 5–95% acetonitrile (0·1% formic acid) at 0·5 ml/min over 20 min. MS/MS spectra were measured on a Bruker amaZon SL ion trap spectrometer. The sequence and retention times of each of the peptides identified are indicated. The presence (✓) or absence (✗) of the peptides in each sample was assessed using extracted ion chromatograms for the indicated transition.

### Identification of the immunogenic component of crude P603

**Figure 3:**
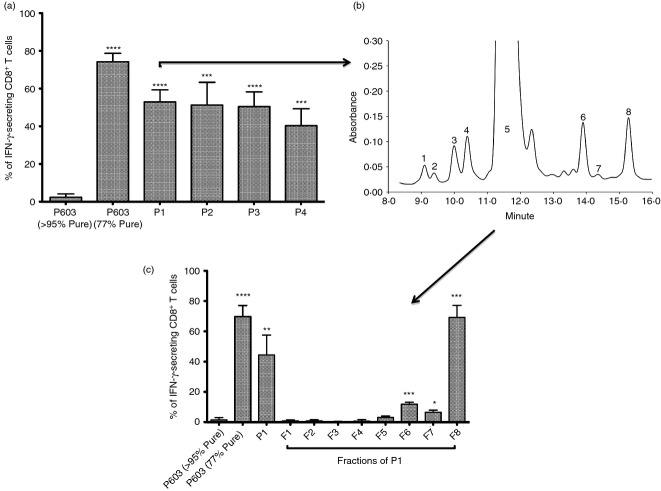
6C5 recognizes a peptide present in fraction 8. (a) CD8^+^ T-cell clone 6C5 was cultured in the presences of various peptide preparations representative of potential C-terminal (GTPT) and N-terminal (LSYF) deletions, as well as C-terminal additions. T cells (1 × 10^5^/tube) were cultured in the presence of T2 or T2 + peptides at a 1 : 1 ratio for 5 hr. Interferon-*γ* (IFN-*γ*) production was assessed through intracellular staining with anti-IFN-*γ*. To facilitate gating, the cultures were also co-stained with anti-CD3 and anti-CD8. Background (T-cells + T2) were subtracted from the data (mean ± SD, *n *= 3). (b) Fractionation was performed by HPLC (Waters 2525 pump and 2996 detector) with a Vydac 218TP 250 × 22 mm column. Peptides were eluted with a binary gradient of 0 min 95% A, 1 min 95% A, 16 min 50% A where solvent A = H_2_O, 0·05% trifluoroacetic acid (TFA) and solvent B = acetonitrile, 0·05% TFA at a flow rate of 22·9 ml/min. The chromatogram shows the absorbance at 220 nm. (c) CD8^+^ T-cell clone 6C5 was cultured in the presence of fractions (F1–F8) generated from the fractionation of the P1 peptide preparation (crude full length 9mer, LLYSFGTPT). T cells (1 × 10^5^/tube) were cultured in the presence of T2 or T2 + peptides at a 1 : 1 ratio for 5 hr. Lymphocytes were gated based on their forward and side scatter profile and then doublet exclusion was performed based on forward scatter height versus forward scatter width. T cells were then gated on CD3^+^ CD8^+^ cells and IFN-*γ* production was assessed through intracellular staining with anti-IFN-*γ*. Background (T-cells + T2) was subtracted from the data (mean ± SD, *n *= 3). Statistical analysis (unpaired two-tailed *t*-test, in comparison to P603 > 95% Pure) was carried out using GraphPad Prism. Significance is indicated by ****< 0·0001, ***0·0001–0·001, **0·001–0·01 and *0·01–0·05.

Based on the earlier observations, the activity of 8mer and 10mer peptides was investigated by deliberate synthesis of a small library of crude peptides. The library consisted of four peptide preparations: (i) full length 9mer (LLYSFGTPT); (ii) enhanced deletions of the *C*-terminal residues (G/T/P/T); (iii) enhanced deletions of the *N*-terminal residues (L/S/Y/F); (iv) 10mer (LLLSYFGTPT, additional *N*-terminal leucine). All four peptide preparations induced similar levels of IFN-*γ* secretion, suggesting that neither a deletion peptide nor the 10mer in isolation were responsible for 6C5 activation (Fig.3a). These results suggested a peptide entity common to all four preparations and the original crude P603 synthesis.

The portion 1 preparation (crude full-length 9mer, LLYSFGTPT) was fractionated by reverse phase HPLC, with fraction collection guided by UV absorbance (Fig.3b). Individual fractions were collected, lyophilized and assayed to determine their capacity to induce T-cell activation. Activity was shown to be associated with a fraction eluting at longer retention time than the principal component (LLSYFGTPT) of the crude peptide preparation (Fig.3c). LCMS analysis of the active fraction revealed a peptide with *m/z* 1053·6 that exhibited an essentially identical fragmentation pattern to the species proposed to contain Tyr(3-*t*Bu), that was identified in the crude P603 (see Supporting information, Fig. S1). The difference in *m/z* between the two species can be accounted for by the differences in C-termini of the peptides, amide versus acid, which arises due to the chemistry of the Rink resin used for preparation of the peptide library.

### Modification of P603

**Figure 4:**
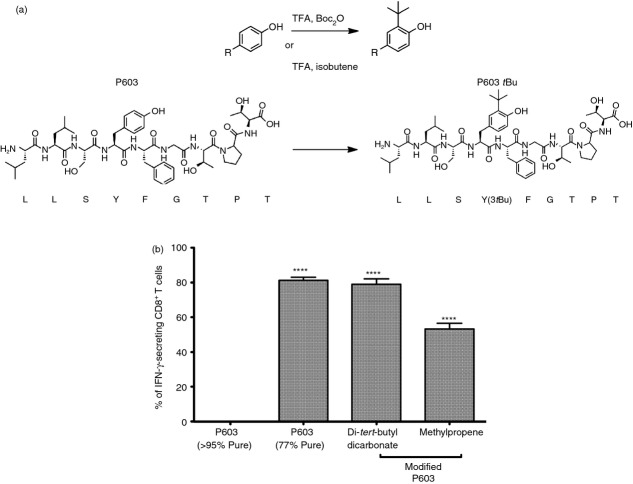
Butylation of the tyrosine residue of p603 confers peptide reactivity. (a) Structure of modified peptide containing the alkylated residue Tyr(3-*t*Bu). Unmodified P603 (> 95% Pure) was reacted with methylpropene and trifluoroacetic acid (TFA), or Boc_2_O and TFA to induce *t*Bu groups on the tyrosine residue. (b) T cells (1 × 10^5^/tube) were cultured in the presence of T2 or T2 + P603 (> 95% Pure, 10 μg/ml), P603 (77% Pure, 10 μg/ml) or the two P603 modified peptides (di-*tert*-butyl dicarbonate and methylpropene) at a 1 : 1 ratio for 5 hr. Lymphocytes were gated based on their forward and side scatter profile and then doublet exclusion was performed based on forward scatter height versus forward scatter width. T cells were then gated on CD3^+^ CD8^+^ cells and IFN-*γ* production was assessed through intracellular staining with anti-IFN-*γ*. Background (T-cells + T2) were subtracted from the data (mean ± SD, *n *= 3). Statistical analysis (unpaired two-tailed *t*-test, in comparison to P603 > 95% Pure) was carried out using Graphpad Prism. Significance was indicated by ****< 0·0001.

To investigate whether a peptide species containing Tyr(3-*t*Bu) was responsible for the observed T-cell activation, the inactive wt P603 (95% pure) preparation was subjected to conditions intended to generate *t*Bu cations. It was predicted that this treatment would re-introduce the alkylated side products and confer activity to a previously inactive wt sequence. Two peptide samples were treated under different conditions, methylpropene with TFA and di-*tert*-butyl dicarbonate with TFA, to mimic the final step of peptide synthesis (Fig.4a). Analysis of the reaction products by HPLC and LCMS demonstrated the introduction of peptides carrying the Tyr(3-*t*Bu). This was visible in both samples, and displayed the same retention time and MS^2^ spectrum as the species observed in the crude P603 (see Supporting information, Fig. S2).

Both of the Tyr(3-*t*Bu) modified preparations were able to induce IFN-*γ* secretion; strongly suggesting that a Tyr(3-*t*Bu) modified peptide was responsible for the activity (Fig.4b).

### P603 Tyr(3-*t*Bu) stimulates the activation of 6C5

**Figure 5:**
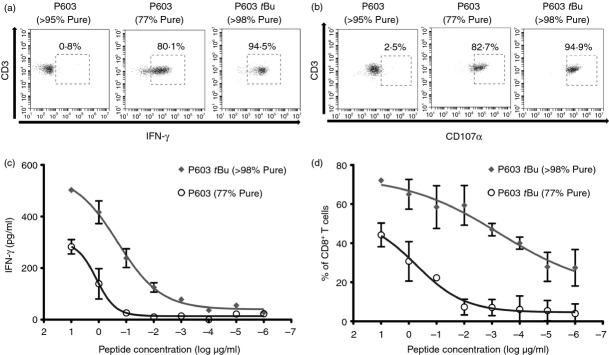
Activation of 6C5 is induced by *t*Bu P603. (a) and (b) Representative sample of flow cytometry analysis (three independent experiments) of intracellular staining of interferon-*γ* (IFN-*γ*) and surface staining of CD107*α*, respectively. T cells (1 × 10^5^/tube) were cultured in the presence of T2 or T2 + P603 (> 95% Pure, 10 μg/ml), P603 (77% Pure, 10 μg/ml) or P603 *t*Bu (> 98% Pure, 10 μg/ml) at a 1 : 1 ratio for 5 hr. IFN-*γ* production was assessed through intracellular staining with anti-IFN-*γ* and CD107*α* expression through surface staining with anti-CD107*α*. To facilitate gating, the cultures were also co-stained with anti-CD3 and anti-CD8 (mean ± SD of duplicates, *n *= 3). Statistical analysis (unpaired two-tailed *t*-test) was carried out using GraphPad Prism. (c) 5 × 10^5^ T2 cells were pulsed with varying concentrations of P603 (77% Pure, 10 μg/ml to 1 × 10^−6^ μg/ml) and P603 *t*Bu (> 98% Pure, 10 μg/ml to 1 × 10^−6^ μg/ml). After 1 hr, unbound peptide was washed off and the pulsed T2 cells were cultured with 1 × 10^5^ 6C5 CD8^+^ T cells at a 1 : 1 ratio. Cell-free supernatant was harvested and tested for the presence of IFN-*γ* by ELISA (mean ± SD, *n *= 3). (d) 1 × 10^5^ 6C5 CD8^+^ T-cells were cultured at 1 : 1 ratio with T2 cells in the presence of varying concentrations of P603 (77% Pure, 10 μg/ml to 1 × 10^−6^ μg/ml) and P603 *t*Bu (> 98% Pure, 10 μg/ml to 1 × 10^−6^ μg/ml) for 5 hr. Lymphocytes were gated based on their forward and side scatter profile and then doublet exclusion was performed based on forward scatter height versus forward scatter width. T cells were then gated on CD3^+^ CD8^+^ cells and changes in the surface expression of CD107*α* were determined through culturing the cells in the presences of anti-CD107*α* (mean ± SD, *n* = 3). The EC_50_ value was calculated using the fitted curve using Graphpad Prism.

To confirm P603 Tyr(3-*t*Bu) was responsible for the activation of 6C5, a highly purified (> 98%) peptide preparation was obtained from a commercial supplier (PolyPeptide Group). This peptide was able to stimulate robust and highly significant (*P* < 0·0001) IFN-*γ* and CD107*α* responses in 6C5. However, the unmodified wt P603 (95% pure) failed to induce T-cell activation in the same assays (Fig.5a,b).

Peptide dose–response experiments were performed to validate the activity of P603 Tyr(3-*t*Bu) and to compare the activity of this peptide against the original crude (77% pure) P603 preparation. P603 Tyr(3-*t*Bu) (> 98% pure) was more potent than P603 (77% pure) as determined by comparison of the calculated EC_50_ values (Fig.5c,d). Using IFN-*γ* production as a functional read-out, the EC_50_ values were 0·2 μm for P603 Tyr(3-*t*Bu) (> 98% pure) and 1·4 μm for P603 (77% pure). By contrast using CD107*α* expression as a read-out, the EC_50_ values were 0·52 nm for P603 Tyr(3-*t*Bu) (> 98% pure) and 610 nm for P603 (77% pure). For both assays the highly purified P603 Tyr(3-*t*Bu) was recognized with greater activity (1–3 logs) than the crude P603 (77% pure). These results would be predicted, as the Tyr(3-*t*Bu) modified form of the peptide was found by HPLC to comprise < 1% of the crude P603 (77% pure) preparation.

**Figure 6:**
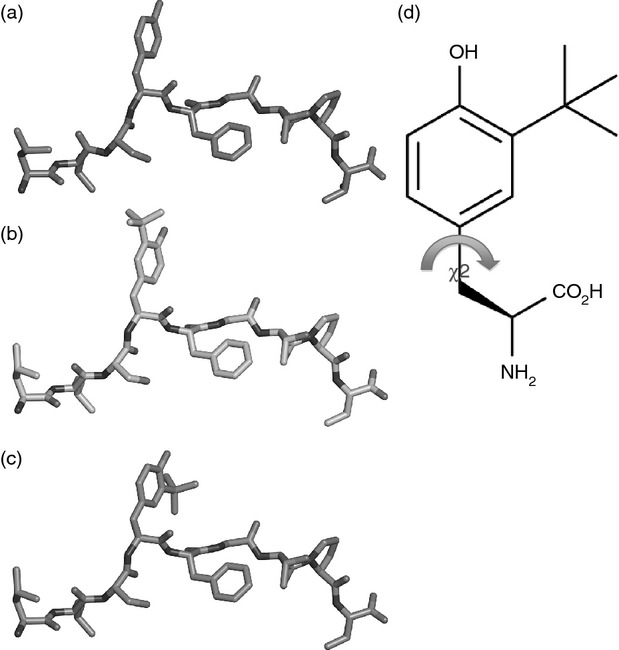
Peptide:MHC modelling of unmodified and Tyr(3-*t*Bu) modified peptides. Computer models illustrating the conformation of peptides bound to HLA-A*0201 were generated based on entry 4I4W from the PDB. The HLA-A*0201 structure has been removed for clarity. COOT was used to modify the sequence of the peptide to be LLSYFGTPT. (a) The resulting modified peptide is capable of adopting two possible conformations (b) and (c) due to the rotation of the aromatic ring about its axis (d).

**Figure 7:**
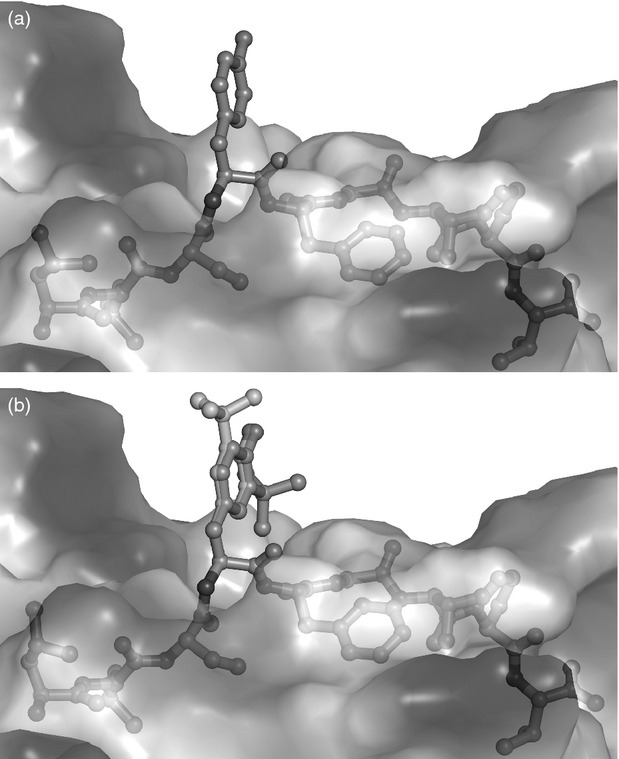
Peptide:MHC modelling of unmodified and Tyr(3-*t*Bu) modified peptide bound to HLA-A*0201. Computer models of Peptide:HLA*0201 complexes were generated using entry 4I4W from the PDB. COOT was used to modify the sequence of the peptide to be LLSYFGTPT. (a) and (b) show that for both peptides the anchor residues at either end of the peptide are well buried in the groove. However, the presence of the *t*Bu-modified tyrosine results in the extension of the side chain out of the MHC and towards the T-cell receptor, which is not observed with the wild-type peptide.

Collectively these findings confirm that the activation of 6C5 is due to the modified P603 [LLSY(3-*t*Bu)FGTPT] rather than the wt P603 peptide (LLSYFGTPT). Molecular modelling of these two peptides indicates that the presence of the Tyr(3-*t*Bu) results in an extension of the tyrosine side chain out of the MHC towards the TCR (Fig.7a,b). Using the model we have predicted that the presence of the Tyr(3-*t*Bu) could result in a peptide with the capacity to adopt two different conformations, due to the rotation of the aryl ring (Fig.6b–d). As the presence of the Tyr(3-*t*Bu) does not appear to affect the peptide conformation or the position of the MHC anchor residue, we predict that the discrimination between the modified and unmodified peptides occurs largely at the level of the TCR. Peptide binding was assessed by the up-regulation of HLA-A*0201 expression on T2 cells. Neither the modified or unmodified peptide were able to significantly up-regulate HLA-A*0201 in comparison to the positive control MART1 peptide, suggesting poor or weak binding under the conditions used (see Supporting information, Fig. S3). Nevertheless, the results suggest that the modified peptide does not have better binding to HLA-A*0201 than the unmodified peptide. This supports our hypothesis that the primary effect of the Tyr(3-*t*Bu) modification is to alter interactions with the TCR.

## Discussion

Minor modifications of antigenic peptides have been shown to have profound positive and negative effects on peptide recognition. These modifications can vary from naturally occurring post-translation modifications^15^ to specific synthetic alterations of the peptide sequence and/or individual amino acids.^4^,^16^ These changes can alter MHC binding; the three-dimensional tertiary peptide structure and the TCR contact residues of the peptides, and so alter peptide immunogenicity.

We describe the novel discovery of a human CD8^+^ T-cell clone that recognizes a *t*Bu-modified peptide LLSY(3-*t*Bu)FGTPT while showing no recognition of the wt peptide LLSYFGTPT. This modification appears to have no effect on the potential structure of the MHC:peptide complex (Fig.7a,b). But it does have a profound effect on T-cell recognition. Similarly it has been observed that a minor change in tyrosine, the conversion of tyrosine to nitrotyrosine, can abrogate peptide recognition in both CD4^+^ T cells^17^ and CD8^+^ T cells.^18^

Peptides containing Tyr(3-*t*Bu) are synthetic entities and do not occur in nature, so it is unclear how CD8^+^ T cells with specificity for Tyr(3-*t*Bu) peptides could arise. There are two possibilities for the origin of T-cell clone 6C5. First, it could be a memory T-cell generated *in vivo* against a naturally occurring epitope that is able to cross-react on Tyr(3-*t*Bu) peptides. Theoretically, a different, naturally occurring residue that possesses an extended side chain could have the correct dimensions to allow for TCR contact. This could be tested by using large peptide libraries to establish the amino acid preference of 6C5 for each position in the peptide.^19^ Second, the clone could have been generated from naive T cells primed against P603 *t*Bu *in vitro*. It is possible that such naive T cells could have been activated and expanded to functionally detectable levels during the 5-week culturing period.^20^

The identification of P603 Tyr(3-*t*Bu) resulted from the detection of a minor peptide contaminant (< 0·05%) present within a crude pool of synthetic peptides. Historically, synthetic peptide pools have been successfully used to generate peptide-specific T-cell responses and clones.^8^ However, one of the major drawbacks associated with this process is the inability to produce completely pure peptide preparations. Commercially produced peptides are commonly subject to stringent purification and quality checking processes, such as reverse-phase HPLC and mass spectrometry. Despite these efforts, contaminants can still be present within the final peptide preparations, potentially causing misleading results.^21^–^24^ This highlights the potential risk that the presence of traces of highly immunogenic contaminating peptides could result in misleading assessment of clinical responses to peptide vaccines. Typically clinical grade peptides used during *in vivo* human vaccine trials range in purity from 95 to ≥ 98%.^25^–^31^ As the contaminant report here constituted < 1% of the original P603 crude peptide preparation and < 0·05% of the original 23-peptide mixture it is conceivable that minute, but highly immunogenic peptide species may be present within clinical grade peptide vaccines. This could lead to the induction of inappropriate responses in T cells with the wrong specificity or even cause adverse reactions.

We believe that the *t*Bu-modified peptide identified in this report was generated during the peptide synthesis process; most likely due to the alkylation of tyrosine by *t*Bu cations under peptide de-protection conditions. This reaction has been reported during model de-protection conditions^32^ and for preparative scale synthesis^33^ of Tyr(3-*t*Bu), therefore the observation of a peptide containing Tyr(3-*t*Bu) as a minor constituent of the crude P603 preparation is not surprising.

In conclusion, the data presented here highlight some issues with interpreting data obtained when using synthetic peptide mixtures. Additionally we have reported that the chemical modification of a single amino acid, Tyr→Tyr(3-*t*Bu), can completely dictate activation of the CD8^+^ T-cell clone 6C5. This peptide modification appears to exert its primary effect via interaction with the TCR rather than influencing the conformation of the peptide within the HLA binding site. These findings emphasize the specificity and sensitivity of TCR and their ability to discriminate between peptides that have an identical primary amino sequence but differ by a single chemical modification.

## Supporting Information

**Figure S1.** Liquid chromatography mass spectrometry analysis of purified and crude P603 peptide samples.

**Figure S2.** MS^2^ spectra of Tyr(3-*t*Bu) containing peptides.

**Figure S3.** Peptide binding to HLA-A2.

## References

[b1] Kawakami Y, Eliyahu S, Jennings C (1995). Recognition of multiple epitopes in the human melanoma antigen gp100 by tumor-infiltrating T lymphocytes associated with *in vivo* tumor regression. J Immunol.

[b2] Rosenberg SA, Yang JC, Schwartzentruber DJ (1998). Immunologic and therapeutic evaluation of a synthetic peptide vaccine for the treatment of patients with metastatic melanoma. Nat Med.

[b3] Brinckerhoff LH, Kalashnikov VV, Thompson LW, Yamshchikov GV, Pierce RA, Galavotti HS, Engelhard VH, Slingluff CL (1999). Terminal modifications inhibit proteolytic degradation of an immunogenic mart-127-35 peptide: Implications for peptide vaccines. Int J Cancer.

[b4] Valmori D, Fonteneau JF, Lizana CM (1998). Enhanced generation of specific tumor-reactive CTL *in vitro* by selected Melan-A/MART-1 immunodominant peptide analogues. J Immunol.

[b5] Kern F, Faulhaber N, Frömmel C (2000). Analysis of CD8 T cell reactivity to cytomegalovirus using protein-spanning pools of overlapping pentadecapeptides. Eur J Immunol.

[b6] Gavioli R, Kurilla MG, de Campos-Lima PO, Wallace LE, Dolcetti R, Murray RJ, Rickinson AB, Masucci MG (1993). Multiple HLA A11-restricted cytotoxic T-lymphocyte epitopes of different immunogenicities in the Epstein–Barr virus-encoded nuclear antigen 4. J Virol.

[b7] Smith KL, Tristram A, Gallagher KM, Fiander AN, Man S (2005). Epitope specificity and longevity of a vaccine-induced human T cell response against HPV18. Int Immunol.

[b8] Nunes CT, Miners KL, Dolton G, Pepper C, Fegan C, Mason MD, Man S (2011). A novel tumor antigen derived from enhanced degradation of bax protein in human cancers. Cancer Res.

[b9] Klenerman P, Cerundolo V, Dunbar PR (2002). Tracking T cells with tetramers: new tales from new tools. Nat Rev Immunol.

[b10] Wong R, Pepper C, Brennan P, Nagorsen D, Man S, Fegan C (2013). Blinatumomab induces autologous T-cell killing of chronic lymphocytic leukemia cells. Haematologica.

[b11] Wellings DA, Atherton E (1996). Standard FMOC protocols. Methods Enzymol.

[b12] Ekeruche-Makinde J, Miles JJ, van den Berg HA (2013). Peptide length determines the outcome of TCR/peptide-MHCI engagement. Blood.

[b13] Emsley P, Cowtan K (2004). Coot: model-building tools for molecular graphics. Acta Crystallogr D Biol Crystallogr.

[b14] Lebedev AA, Young P, Isupov MN, Moroz OV, Vagin AA, Murshudov GN (2012). JLigand: a graphical tool for the CCP4 template-restraint library. Acta Crystallogr D Biol Crystallogr.

[b15] Engelhard VH, Altrich-Vanlith M, Ostankovitch M, Zarling AL (2006). Post-translational modifications of naturally processed MHC-binding epitopes. Curr Opin Immunol.

[b16] Parkhurst MR, Salgaller ML, Southwood S, Robbins PF, Sette A, Rosenberg SA, Kawakami Y (1996). Improved induction of melanoma-reactive CTL with peptides from the melanoma antigen gp100 modified at HLA-A* 0201-binding residues. J Immunol.

[b17] Birnboim HC, Lemay A-M, Lam DKY, Goldstein R, Webb JR (2003). Cutting edge: MHC class II-restricted peptides containing the inflammation-associated marker 3-nitrotyrosine evade central tolerance and elicit a robust cell-mediated immune response. J Immunol.

[b18] Hardy LL, Wick DA, Webb JR (2008). Conversion of tyrosine to the inflammation-associated analog 3′-nitrotyrosine at either TCR-or MHC-contact positions can profoundly affect recognition of the MHC class I-restricted epitope of lymphocytic choriomeningitis virus glycoprotein 33 by CD8 T cells. J Immunol.

[b19] Wooldridge L, Ekeruche-Makinde J, van den Berg HA (2012). A single autoimmune T cell receptor recognizes more than a million different peptides. J Biol Chem.

[b20] Cerny A, Fowler P, Brothers MA, Houghton M, Schlicht HJ, Chisari FV (1995). Induction *in vitro* of a primary human antiviral cytotoxic T cell response. Eur J Immunol.

[b21] Brezar V, Culina S, Østerbye T (2011). T cells recognizing a peptide contaminant undetectable by mass spectrometry. PLoS ONE.

[b22] Mannering SI, Purcell AW, Honeyman MC, McCluskey J, Harrison LC (2003). Human T-cells recognise N-terminally Fmoc-modified peptide. Vaccine.

[b23] Currier JR, Galley LM, Wenschuh H (2008). Peptide impurities in commercial synthetic peptides and their implications for vaccine trial assessment. Clin Vaccine Immunol.

[b24] De Spiegeleer B, Vergote V, Pezeshki A, Peremans K, Burvenich C (2008). Impurity profiling quality control testing of synthetic peptides using liquid chromatography-photodiode array-fluorescence and liquid chromatography-electrospray ionization-mass spectrometry: the obestatin case. Anal Biochem.

[b25] Iinuma H, Fukushima R, Inaba T (2014). Phase I clinical study of multiple epitope peptide vaccine combined with chemoradiation therapy in esophageal cancer patients. J Transl Med.

[b26] Betancourt AA, Delgado CAG, Estévez ZC (2007). Phase I clinical trial in healthy adults of a nasal vaccine candidate containing recombinant hepatitis B surface and core antigens. Int J Infect Dis.

[b27] Hueman MT, Dehqanzada ZA, Novak TE (2005). Phase I Clinical trial of a HER-2/neu peptide (E75) vaccine for the prevention of prostate-specific antigen recurrence in high-risk prostate cancer patients. Clin Cancer Res.

[b28] Okuno K, Sugiura F, Hida J, Tokoro T, Ishimaru E, Sukegawa Y, Ueda K (2010). Phase I clinical trial of a novel peptide vaccine in combination with UFT/LV for metastatic colorectal cancer. Exp Ther Med.

[b29] Aruga A, Takeshita N, Kotera Y, Okuyama R, Matsushita N, Ohta T, Kazuyoshi T, Yamamoto M (2014). Phase I clinical trial of multiple-peptide vaccination for patients with advanced biliary tract cancer. J Transl Med.

[b30] Carreno BM, Becker-Hapak M, Huang A (2013). IL-12p70-producing patient DC vaccine elicits Tc1-polarized immunity. J Clin Invest.

[b31] Muderspach L, Wilczynski S, Roman L (2000). A phase I trial of a human papillomavirus (HPV) peptide vaccine for women with high-grade cervical and vulvar intraepithelial neoplasia who are HPV 16 positive. Clin Cancer Res.

[b32] Lundt BF, Johansen NL, Markussen J (1979). Formation and synthesis of 3′-t-butyltyrosine. Int J Pept Protein Res.

[b33] Taka N, Matsuoka H, Sato T (2009). Discovery of novel motilin antagonists: conversion of tetrapeptide leads to orally available peptidomimetics. Bioorg Med Chem Lett.

