# Supplementary material for: CD8+ T-cell recognition of a synthetic epitope formed by t-butyl modification
Source: Immunology. 2015 Mar;144(3):495–505. doi: 10.1111/imm.12398 (PMC4557686; doi:10.1111/imm.12398)
Supplement: Supplementary file 1 [file imm0144-0495-sd1.ppt]

## Slide 1
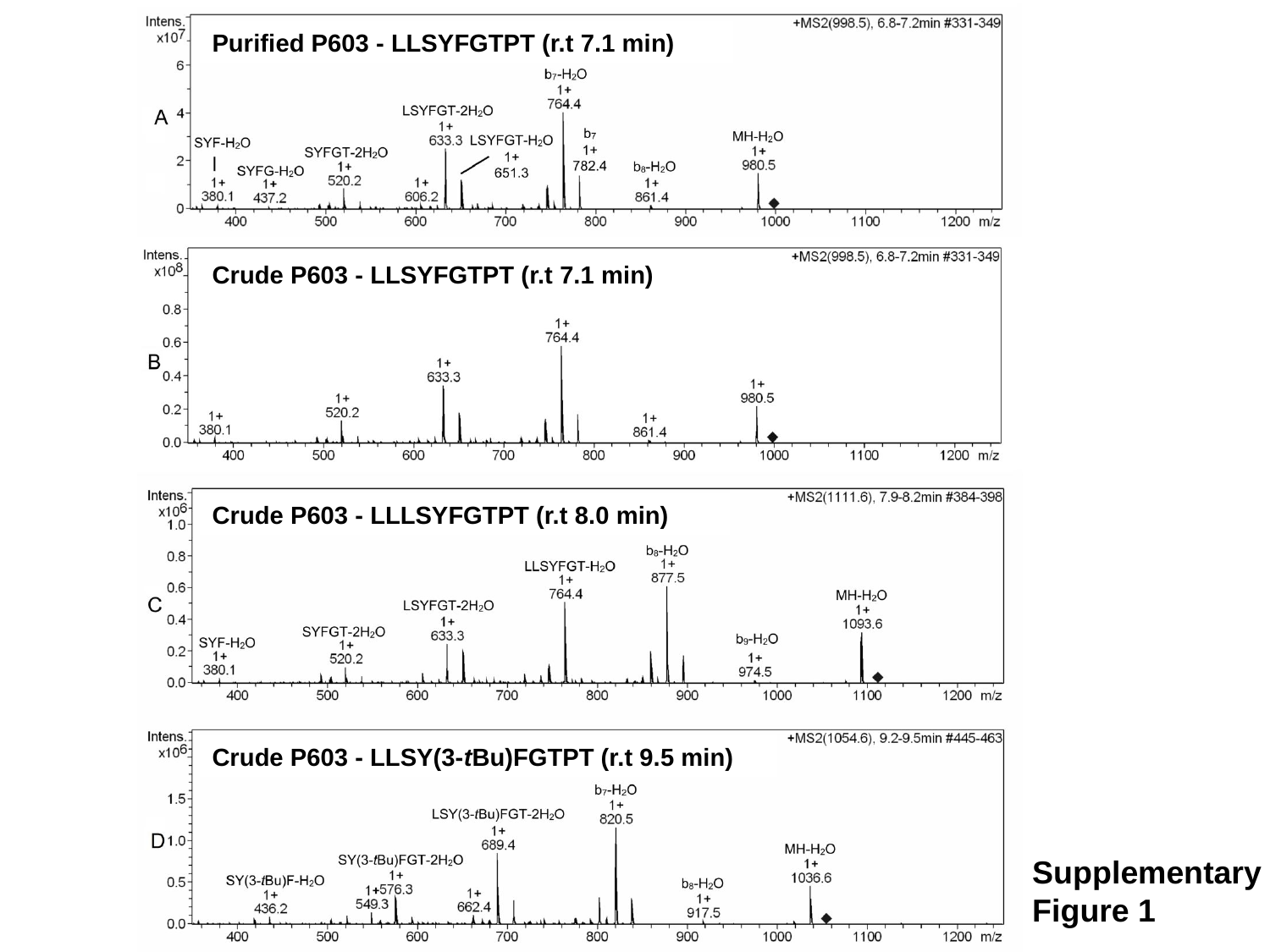

Purified P603 - LLSYFGTPT (r.t 7.1 min)
Crude P603 - LLSYFGTPT (r.t 7.1 min)
Crude P603 - LLLSYFGTPT (r.t 8.0 min)
Crude P603 - LLSY(3-tBu)FGTPT (r.t 9.5 min)
Supplementary Figure 1
