# Supplementary material for: CD8+ T-cell recognition of a synthetic epitope formed by t-butyl modification
Source: Immunology. 2015 Mar;144(3):495–505. doi: 10.1111/imm.12398 (PMC4557686; doi:10.1111/imm.12398)
Supplement: Supplementary file 2 [file imm0144-0495-sd2.ppt]

## Slide 1
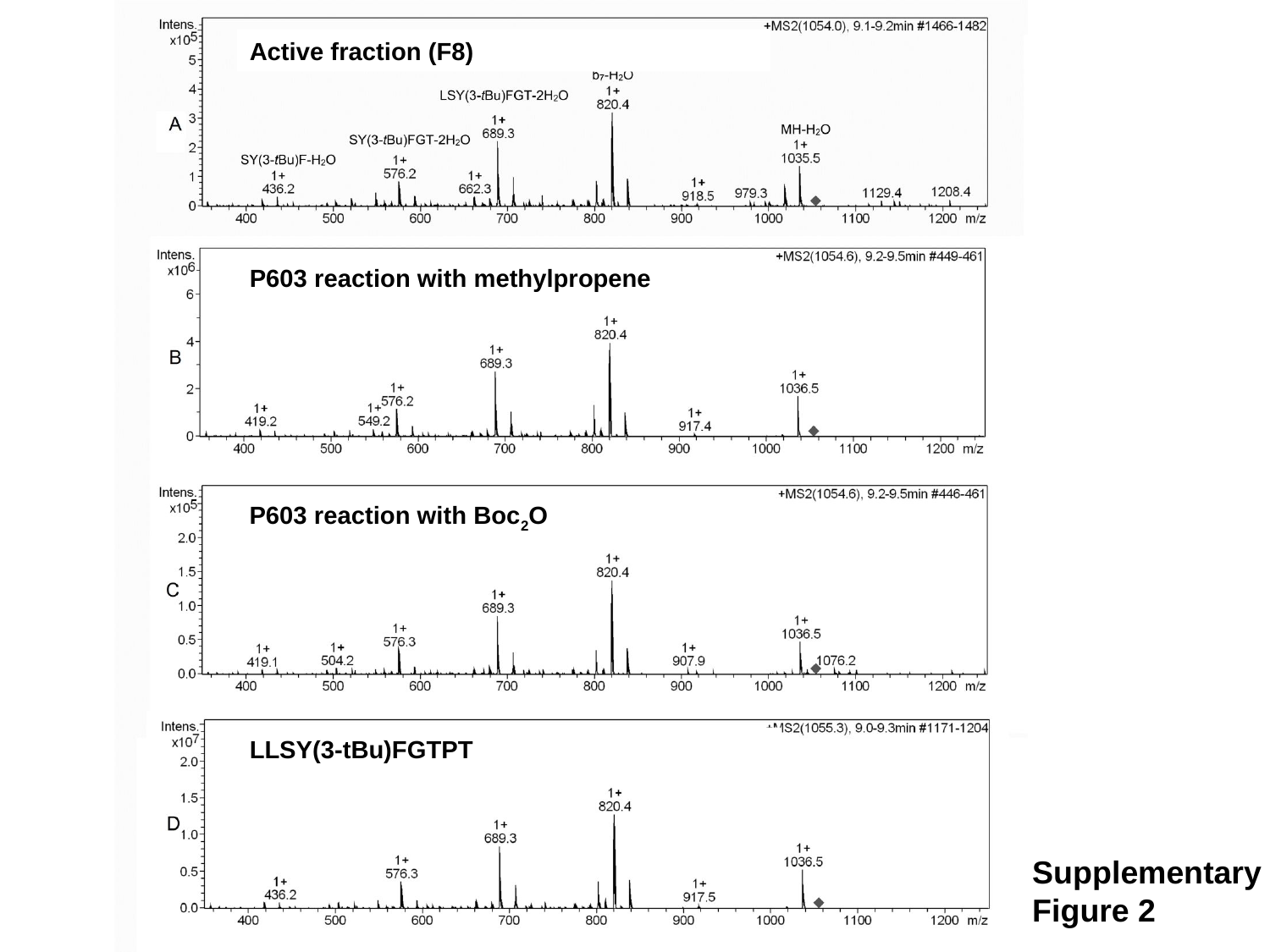

Active fraction (F8)
P603 reaction with methylpropene
P603 reaction with Boc2O
LLSY(3-tBu)FGTPT
Supplementary Figure 2
