# Supplementary figures and images for: CD8+ T-cell recognition of a synthetic epitope formed by t-butyl modification
Source: Immunology. 2015 Mar;144(3):495–505. doi: 10.1111/imm.12398 (PMC4557686; doi:10.1111/imm.12398)

## Slide 1
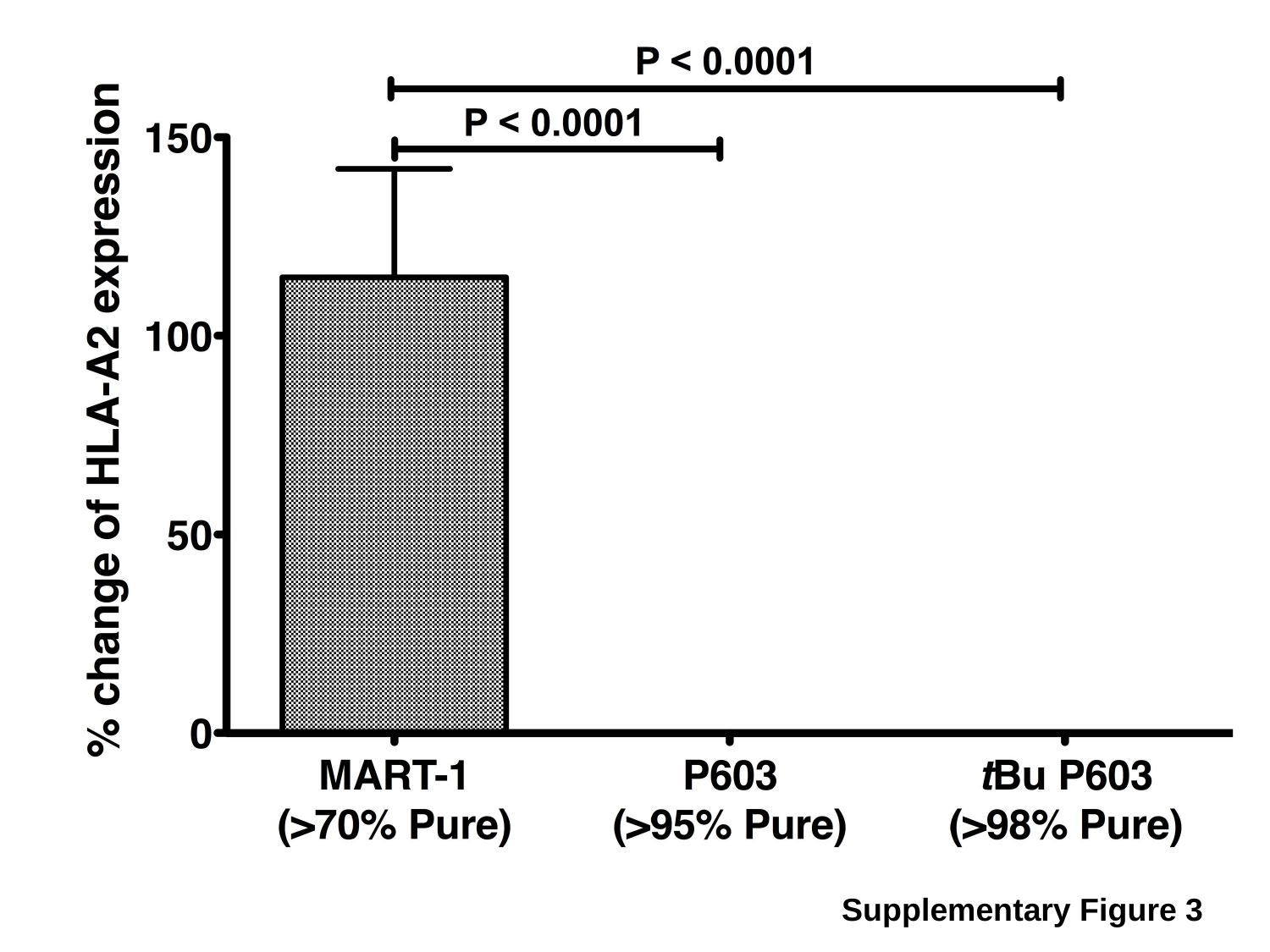

Supplementary Figure 3

Supplement: Supplementary file 3 [file imm0144-0495-sd3.ppt]
