# Supplementary material for: CD8+ T-cell recognition of a synthetic epitope formed by t-butyl modification
Source: Immunology. 2015 Mar;144(3):495–505. doi: 10.1111/imm.12398 (PMC4557686; doi:10.1111/imm.12398)
Supplement: Supplementary file 4 [file imm0144-0495-sd4.docx]

**Figure S1. LCMS analysis of purified and crude P603 peptide samples**. Chromatography was performed on an Ace Excel 2 100 × 2.1 mm column (C18), eluting with a linear gradient of 5 – 95 % acetonitrile (0.1 % formic acid) at 0.5 ml/min over 20 minutes. MS^2^ spectra are shown with the *m*/*z* of the precursor ion indicated with a diamond, ◆. **(A)** Purified p603, r.t. 7.1 min, precursor *m*/*z* 998.5, LLSYFGTPT. **(B)** Crude p603, r.t. 7.1 min, precursor *m*/*z* 998.5, LLSYFGTPT. **(C)** Crude p603, r.t. 8.0 min, precursor *m*/*z* 1111.6, LLLSYFGTPT. **(D)** Crude p603, r.t. 9.5 min, precursor *m*/*z* 1054.6, LLSY(3-*t*Bu)FGTPT.

**Figure S2. MS^2^ spectra of Tyr(3-*t*Bu) containing peptides**. The *m*/*z* of the precursor ion is indicated with a diamond, ◆. **(A)** Active fraction (F8) from HPLC fractionation. **(B)** P603 reaction with methylpropene. **(C)** P603 reaction with Boc_2_O. **(D)** Commercial sample of purified LLSY(3-*t*Bu)FGTPT.

**Figure 3. Peptide binding to HLA-A2**. Binding was determined by flow cytometric measurement of HLA-A2 expression on the TAP deficient T2 cell line. Peptides were added to FAC tubes containing T2 cells and after overnight culture cells were stained with anti-human HLA-A2.1 antibody. T2 cells were gated based on their forward and side scatter profile and then doublet exclusion was preformed based on forward scatter height versus forward scatter width. HLA-A2 expression was assess with HLA-A2 antibody. HLA-A2 expression was quantified as a percentage increase according to the formula: % Increase= [(mean fluorescence with peptide – mean fluorescence without peptide)/ mean fluorescence without peptide x100]. A known HLA-A2 binding peptide, Melanoma MART-1, was included as a positive control.
